# Anti-Tat Immunity in HIV-1 Infection: Effects of Naturally Occurring and Vaccine-Induced Antibodies Against Tat on the Course of the Disease

**DOI:** 10.3390/vaccines7030099

**Published:** 2019-08-26

**Authors:** Aurelio Cafaro, Antonella Tripiciano, Orietta Picconi, Cecilia Sgadari, Sonia Moretti, Stefano Buttò, Paolo Monini, Barbara Ensoli

**Affiliations:** National HIV/AIDS Research Center, Istituto Superiore di Sanità, Rome 00161, Italy

**Keywords:** HIV-1 Tat, anti-Tat antibodies, natural vs. vaccine-induced antibody response, crossclade antibodies, HIV-1 vaccine development, HIV-1 Tat therapeutic vaccine, HIV reservoir, cART intensification, functional cure, perspective for clinical implications

## Abstract

HIV-1 Tat is an essential protein in the virus life cycle, which is required for virus gene expression and replication. Most Tat that is produced during infection is released extracellularly and it plays a key role in HIV pathogenesis, including residual disease upon combination antiretroviral therapy (cART). Here, we review epidemiological and experimental evidence showing that antibodies against HIV-1 Tat, infrequently occurring in natural infection, play a protective role against disease progression, and that vaccine targeting Tat can intensify cART. In fact, Tat vaccination of subjects on suppressive cART in Italy and South Africa promoted immune restoration, including CD4+ T-cell increase in low immunological responders, and a reduction of proviral DNA even after six years of cART, when both CD4+ T-cell gain and DNA decay have reached a plateau. Of note, DNA decay was predicted by the neutralization of Tat-mediated entry of Env into dendritic cells by anti-Tat antibodies, which were cross-clade binding and neutralizing. Anti-Tat cellular immunity also contributed to the DNA decay. Based on these data, we propose the Tat therapeutic vaccine as a pathogenesis-driven intervention that effectively intensifies cART and it may lead to a functional cure, providing new perspectives and opportunities also for prevention and virus eradication strategies.

## 1. Introduction

An HIV vaccine is still the most valuable and cost-effective intervention to end the HIV/AIDS pandemic [1]. We focused on the HIV-1 Tat (TransActivator of Transcription) protein as an optimal pathogenetic vaccine target because of the inherent difficulties in developing vaccine strategies capable of inducing cross clade broadly neutralizing antibodies (bNAbs) against HIV-1 Env [2]. This strategy stems from the results of epidemiological and basic research studies on the role of Tat and anti-Tat immunity in the course of HIV infection.

Here, we briefly review epidemiological and experimental evidence showing the role of Tat in the virus life cycle, with particular emphasis on extracellular Tat and on its effects on both the virus and the immune system. Conversely, we examine and discuss data on the protective effect of anti-Tat antibodies (Abs), naturally occurring or vaccine-induced, on the course of the disease including in suppressive cART. Further, we will discuss evidence on the role of anti-Tat Abs in blocking the formation of the Tat/Env complex that allows for Tat-mediated HIV entry through the RGD (arginyl-glycyl-aspartic acid tripeptide) binding integrins α5β1, αvβ3, and αvβ5, and it enhances the infection of cells expressing these integrins, such as dendritic cells (DCs). This is a novel path of virus entry and cell infection, likely increasing HIV acquisition, as indicated by results in monkeys’ studies, and most likely contributes to reservoir generation and maintenance. Finally, we examine the effects of anti-Tat Abs in accelerating the decay of the CD4+ T-cell HIV reservoir under long-term cART. Taken together, the results from the studies described here suggest that targeting HIV-1 Tat, a very early HIV protein, may represent a valuable strategy to intensify cART to achieve HIV functional cure or eradication. Similarly, a vaccine targeting the Tat/Env complex may represent a novel strategy to prevent HIV acquisition, as will be discussed below.

## 2. Role of Tat in the Virus Life Cycle

**Figure 1:**
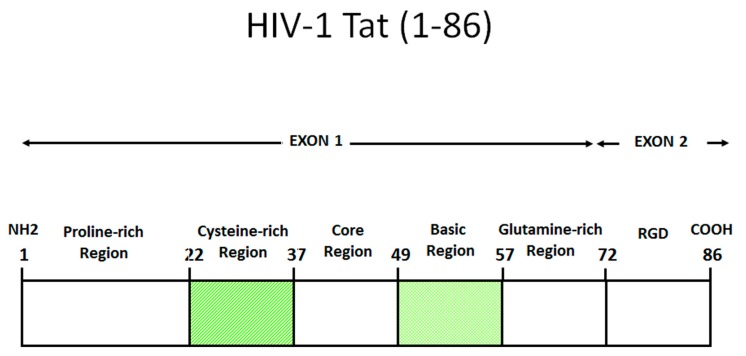
Functional domains of HIV-1 Tat (HXB2). Amino acid numbering according to the IIIB/Lai sequence. Tat can be divided into six domains: a proline-rich acidic N-terminus (aa 1–21), a cysteine-rich region (aa 22–37), a hydrophobic core region (aa 38–48), an arginine-rich basic domain (aa 49–57), a glutamin-rich region (aa 60–76) and a C-terminal domain containing the RGD sequence, recognized by RGD-binding integrins. Shown is the 86 aa long form of Tat, commonly found in cell lines and used experimentally. However, in field isolates the 101 aa long form of Tat is mostly found [8].

Tat is generated in two forms through alternative splicing. The first form is encoded by the multiply spliced two-exon transcript and it varies in length between 86 and 101 amino acids (aa), depending on the viral isolate. The other is encoded by a singly spliced one-exon transcript and it is 72 aa long. Both Tat variants transactivate the LTR efficiently, but the two-exon Tat appears to exert additional effects on the infected cell, which affect cytoskeleton structure and function [3], delay Fas-mediated apoptosis [4], and reduce the triggering of innate and adaptive immune responses, such as the downregulation of interferon-stimulated genes and MHC class-I and II molecules gene expression in antigen presenting cells [5,6,7]. Although both the one- and the two- exons forms of Tat are produced and functional, the latter is the form prevalently found in vivo (101 aa) and in vitro (86 aa) [8]. As shown in Figure 1, Tat contains six domains: a proline-rich acidic N-terminus (aa 1–21), a cysteine-rich region (aa 22–37), a hydrophobic core region (aa 38–48), an arginine-rich basic domain (aa 49–57), a glutamin-rich region (aa 60–76), and a C-terminal domain containing the RGD sequence, as recognized by RGD-binding integrins. Of note, Tat is largely unstructured and very flexible, a property that permits interactions with numerous partners, as reported [8,9,10,11,12]. Unless differently stated, thereafter the terms Tat refers to the two-exon Tat, particularly to the shortest form of 86 aa in length.

Although prominently known for its role in promoting transcription elongation, Tat participates and regulates several aspects of the HIV gene transcription program (Table 1), both in the acute phase of infection and reactivation, irrespective of cART [13,14,15,16,17].

**Figure 2:**
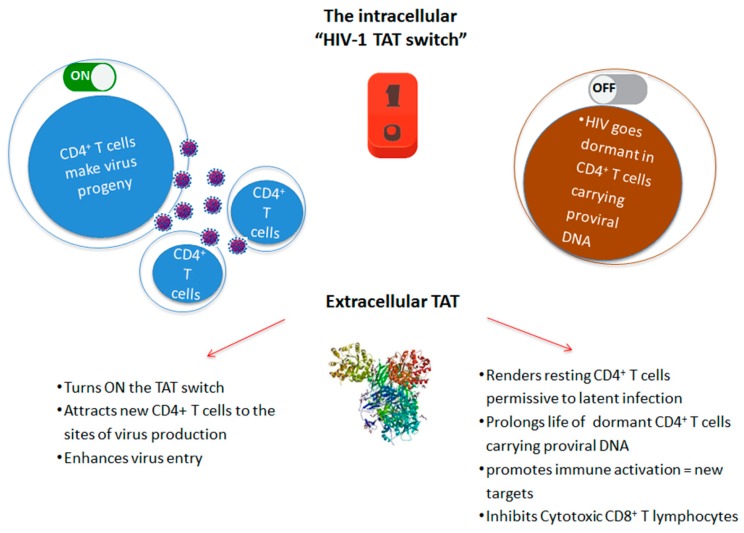
Role of intracellular and extracellular HIV-1 Tat in the virus life cycle. Tat drives both productive infection and establishment of latent virus reservoirs by increasing permissivity and half-life of resting CD4+ T cells harboring HIV proviral DNA.

In particular, Tat is central in determining the cell fate toward HIV productive or latent infection, and it acts as a “stochastic switch” (Figure 2), which appears to be uncoupled and independent from environmental stimuli [13,17]. In fact, even strong cell activating stimuli cause the (sporadic) reactivation of only a fraction of replication-competent proviruses [28,29], which is most likely due to the role played by the ensuing Tat stochastic circuitry [30]. Noticeably, these data challenge the concept that the so-called shock-and-kill strategy can deterministically reactivate “all” (or even “most of”) latent HIV to eradicate the virus.

The confirmation of the critical role of Tat in the virus life cycle comes also from “Block-and-Lock’” studies with Tat inhibitory/antagonist molecules. In particular, the small molecule didehydro-Cortistatin A (dCA) has been reported to bind the basic region of HIV Tat, thus preventing its binding to TAR-RNA [31]. As a result, ex vivo viral reactivation from patient CD4+ T cells is blocked [32], and, in vivo, simian immunodeficiency virus (SIV) replication and reactivation are suppressed [33].

## 3. Role of Extracellular Tat

The majority (about 65%) of the Tat protein produced by the infected cell released is extracellularly in the absence of cell death or cell permeability changes by an unconventional, leaderless secretory pathway similar to that used by basic fibroblast growth factor (bFGF) or interleukin-1β (IL-1β) to exit cells [21,34,35,36,37]. In particular, Tat appears to specifically bind the fraction of phosphatidylinositol-(4,5)-bisphosphate [PI(4,5)P2] that is located at the inner leaflet of the plasma membrane [36] and to be released extracellularly by exocytosis [21], with a mechanism still poorly understood [37]. Of importance, plasma membrane perturbation by Tat has been proposed [37] to interfere within several biological processes in which PI(4,5)P2 is involved, such as clathrin-mediated endocytosis [38], phagocytosis [39], or exocytosis [40]. Recent evidence indicates that Tat is also released extracellularly within exosomes [41], which appear to be enriched with small noncoding RNAs containing TAR [42], and their derivatives TAR miRNA [43], which, in turn, have been reported to promote, respectively, inflammation and tumorigenesis, two hallmarks of the residual disease observed in subjects on long-term suppressive cART [43,44].

Upon release, Tat binds heparan sulphate proteoglycans (HSPG) of the extracellular matrix (ECM) through its basic region, and it is detected in tissues of infected individuals [20,21,34,45,46,47]. Extracellular Tat is biologically active and it exerts activities key for acquisition of infection, virus reactivation, and HIV disease maintenance in cART treated individuals [19,20,21,35,36,45,46,47,48,49,50,51,52,53,54,55,56].

Bioactive Tat, which carries an RGD domain in the second exon (aa 79-81), recognizes the RGD binding integrins α5β1, αvβ3, and αvβ5, and it has been shown to target cells expressing these integrin receptors, including DCs [54], macrophages [57], activated endothelial cells (ECs) [20], and lymphocytes [58]. In particular, extracellular Tat enters very selectively and efficiently DCs and activated ECs, and also CD4+ T cells, although this requires a higher concentrations gradient [20,54,55]. In DCs, Tat promotes cell maturation toward a Th-1 polarizing phenotype. It further modulates T cell responses responses [54,55,56,59] and it activates the immunoproteasome, leading to increased antigen processing and presentation, thus contributing to Th-1 cell activation [52,60,61]. Moreover, upon engagement of CD8 T cells, Tat promotes the induction of short living effectors [62,63], which undergo apoptosis upon encountering antigens in the presence of Tat [64]. On CD4+ T cells, the engagement of CD26 by Tat makes lymphocytes unresponsive to recall antigens [65], an immunosuppression that is counteracted by the addition of soluble CD26 to CD4+ T cells from HIV-infected individuals [66]. Extracellular Tat also suppresses T-cell activation by inducing in DCs the expression of indoleamine 2,3 dioxygenase (IDO) [67], a biomarker that is associated with disease progression [68]. Further, Tat harnesses innate immunity, since it binds to Toll-like receptor 4 and activates the expression of cytokines with key immunomodulatory effects and/or capable of activating HIV gene expression [56,69,70,71,72,73,74]. Through these activities, extracellular Tat impairs several arms of the immune response against pathogens, including HIV, a feature that might be key, not only for HIV-driven immune dysregulation/suppression, but, notably, for the maintenance of HIV reservoirs (see below). Moreover, extracellular Tat mimics chemokines [75], attracts CXCR4+ naïve T cells to the site of virus production, induces the expression of both CXCR4 and CCR5 co-receptors [76], and favors the transmission of R5-tropic viruses to neighbour cells [77], which may occur, even in the absence of cell activation [59].

**Figure 3:**
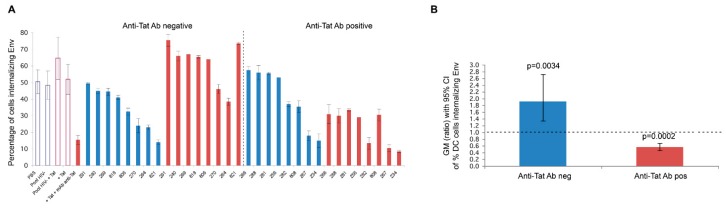
Neutralization of Tat/Env complex entry into monocyte-derived DCs (MDDCs) by sera from HIV-infected individuals. (**A**) Neutralization of trimeric Env ΔV2 entry into MDDCs by sera from HIV-infected subjects in the presence or absence of Tat (0.01 µM) in anti-Tat Ab negative (*n* = 8) and anti-Tat Ab positive (*n* = 8) subjects. The bars represent the percentage of entry of Env alone incubated in buffer (in blue) or with Tat (in red). The percentage of Env positive cells is shown. Data are expressed as the mean with standard deviation of experiments performed in duplicate. The codes of the anti-Tat Ab negative or positive sera are indicated at the bottom of the bars. (**B**) Geometric mean (GM) of the ratio, with 95% confidence interval (CI) of the percentage of MDDCs internalizing Env in the absence (blue bar) or in the presence (red bar) of Tat in anti-Tat Ab negative (*n* = 8) and anti-Tat Ab positive (*n* = 8) subjects. Statistical analysis was performed by the two-tailed Student’s t-test.

Of importance, we recently found that Tat binds the oligomeric form of Env displayed on the virion, thus enabling the virus to enter DCs exploiting an integrin-mediated endocytic pathway [78]. This pathway of entry is an alternative to the canonical Env-dependent C-type lectin endocytic pathway and it leads to enhancement of infection of DCs, which, in turn, very efficiently transmit the infection to T cells [78]. In particular, the cysteine-rich region of Tat binds the Env V3 loop, whereas the Tat RGD sequence remains exposed, which confers to the virus the capability to recognize the integrins α5β1, αvβ3, and αvβ5. V2 loop deletion, which unshields the CCR5 binding region of Env, increases Tat/Env complex stability. As a result, HIV acquisition by relatively poor susceptible cell targets—but important viral reservoirs, such as DCs, and possibly other cell types not expressing the canonical HIV-1 receptors—is greatly increased. By redirecting HIV to the RGD-binding integrins, Tat shields the bound Env oligomer from anti-Env neutralizing Abs (i.e., Abs capable of blocking the C-type lectin receptor entry pathway), as indicated by a novel entry-neutralization assay that evaluates the capability of anti-HIV-1 Abs to block the entry of oligomeric Env into DCs, in the presence or absence of Tat [78]. In fact, in the presence of Tat, sera from HIV-1-infected individuals who are negative for anti-Tat Abs become ineffective at blocking the entry of Env in DCs; however, when anti-Tat Abs are present, the neutralization of Env entry is not only restored, but further enhanced (Figure 3).

**Figure 4:**
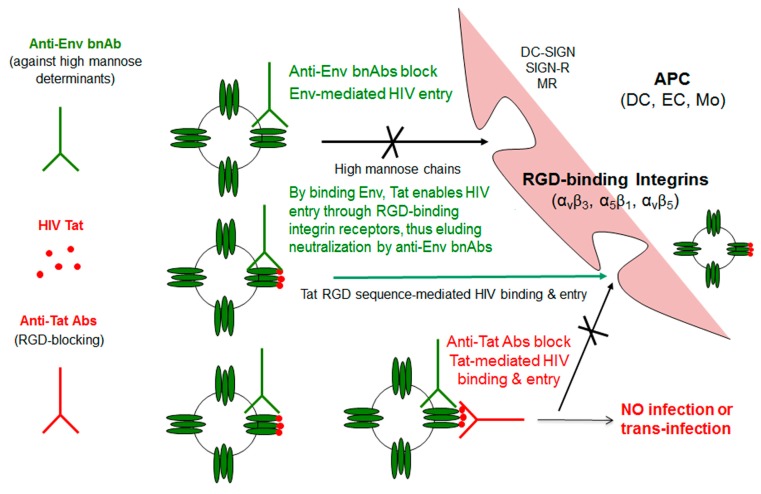
Tat-mediated entry of HIV and role of antibodies against Env or Tat. By binding Tat, HIV acquires the capability of using RGD binding integrins to enter cells, circumventing neutralization by anti-Env Abs and greatly expanding its spreading potential. Anti-Tat Abs effectively counteract this entry pathway. APC: Antigen-presenting cell; DC: Dendritic cell; DC-SIGN: Dendritic cell-specific intercellular adhesion molecule-3-grabbing non-integrin; DC-SIGN-R: DC-SIGN-related; EC: Endothelial cell; Mo: Monocyte/macrophage; MR: Mannose receptor; RGD: Arg-Gly-Asp motif; Tat: Transactivator of transcription.

These data are consistent with the model that is depicted in Figure 4, which shows that extracellular Tat that is released by infected neighbour cells binds to trimeric Env on HIV, decreases the recognition of C-type lectin receptors, and promotes the engagement of RGD-binding integrins, which are expressed by antigen-presenting cells (APCs), such as inflammatory DCs, macrophages (Mo), and ECs that are present at the site of infection. As a result, virions escape anti-Env Abs directed against high mannose determinants and enter target cells upon binding to RGD-binding integrins, a pathway that is blocked by Anti-Tat Abs.

## 4. Role of Extracellular Tat in HIV Reservoir Maintenance and Residual Disease upon Effective cART

Although cART suppresses HIV replication to levels that are undetectable in the peripheral blood, a low-level, intermittent residual plasma viremia (<50 copies per mL), as well as viral “blips” (50–1.000 copies/mL) are detected in most HIV-1-infected patients, even after several years of treatment [79,80]. Residual viremia and blips have been found to be predictive of virus rebound [81,82] and, conceivably, are one of the major sources of persistent immune activation, residual disease, and comorbidities in treated patients. The exact origin of residual viremia is debated [83], but evidence indicates contributions from both the reactivation of latent HIV [84], which is inherently insensitive to cART, and residual virus replication, which is driven, in turn, by low drug penetration in lymphoid tissues [85,86], as well as by drug-resistant cell-to-cell transmission [87]. Accordingly, HIV gene expression is not fully suppressed under cART [88,89,90,91], and Tat has been reported to be produced and released in treated patients ([92,93]. In this regard, our unpublished data indicate that infected lymphocytes that were treated with current antiretrovirals, while blocking productive infection and reducing the level of unspliced and singly-spliced RNA transcripts, continue to express RNA transcripts encoding for Tat, Rev, and Nef, which alters the expression ratio in favor of multispliced transcripts, consistent with reported results [88,89,90,91]. Such residual gene expression also contributes to persistent immune activation and residual disease under cART. Indeed, extracellular Tat crosses the blood brain barrier and promotes the Central Nervous System (CNS) inflammation and T-cell activation [59], which persist, despite treatment intensification with CNS penetrating ART [94]. Thus, virus reactivation and residual replication, both sustaining Tat production and release under cART, are major factors for persistent immune activation and residual disease in treated patients.

In this context, several lines of evidence suggest that extracellular Tat plays a key role in both HIV reactivation and residual replication. In fact, the internalization of extracellular Tat by latently infected cells turns ON the Tat intracellular stochastic switch (Figure 2), as indicated by studies showing that, upon entry into cells, Tat trans-activates the HIV LTR, leading to Tat-defective provirus rescue and latent virus replication [35,49,95,96]. Although infection in sub-optimal drug compartments is highly vulnerable to stochastic extinction, owing to unfavourable levels of virions’ production, our data indicate that the enhancement of HIV infectivity by the Tat/Env complex is particularly significant at low multiplicity of infection [78]. These data suggest that, by releasing Tat, the virus establishes an extracellular positive circuitry at sites of HIV reactivation/residual replication to maximize the chances for propagation.

Evidence also indicates that extracellular Tat exerts several effects to stabilize the HIV latent reservoir. The cells populating the CD4+ T-cell reservoir are mainly memory cells that are cleared by several mechanisms, including cell senescence and stochastic cell death as well as HIV reactivation, which leads to virus-mediated cytopathic effects and cell killing by cytotoxic CD8+ T cells, Ab-dependent cellular cytotoxicity, or Ab-mediated phagocytosis [97,98]. Consequently, cART initiation leads to a prompt and steep decline of HIV latently infected cells that, however, subsequently evens up to a rather stable (quasi-steady state) dynamic level, with a half-life of about 3.6 years [99,100,101,102,103]. The reason for the stability of the HIV reservoir under long-term cART resides in several mechanisms, which includes the intrinsic homeostasis of the immune memory compartment (i.e., the slow cell turn-over and antigen-driven asymmetric cell-division) [104] as well as the clonal/oligoclonal proliferation/expansion of CD4+ T cells carrying competent HIV proviral DNA [29,97,105,106]. Further, in an *ex-vivo* approach, it has been observed that the efficient killing of latently-infected CD4+ T cells during HIV reactivation requires the re-stimulation of autologous HIV-specific cytotoxic T cells (CTLs), pointing to insufficient cell cytotoxicity, even in virologically suppressed patients [107]. Moreover, CD4+ T cells undergoing effector-to-memory transition have been reported to be “primed” for HIV latent infection due to the transient upregulation of HIV co-receptor CCR5 and concomitant gene expression down-regulation, which silences the incoming provirus [108]. Hence, these cells are the most susceptible to latent HIV infection in compartments with low-drug penetration. Notably, the establishment of latent infection in these transitioning cells is inhibited by HIV-specific CTLs [108].

In this regard, extracellular Tat is known to favor the differentiation of naïve CD4+T cells towards the effector-memory phenotype [62], hence generating a pool of cells that are highly prone to latent infection. Moreover, extracellular Tat delays Fas-mediated apoptosis in infected PBMCs [4] and upregulates anti-apoptotic genes, particularly Bcl-2, in various cell types, including PBMCs, which thereby promotes their survival [109]. Thus, extracellular Tat may prolong the half-life of the HIV CD4+ T-cell reservoir. Finally, the extracellular Tat inhibits the activation and cytotoxic activity of CD8+ T cells, irrespective of their antigenic specificity [64] (Figure 2).

Thus, Tat appears to play a critical role in all key aspects of the virus life cycle. In fact, it promotes, on the one hand, productive infection and virus spreading, on the other hand, latency establishment and reservoir maintenance and replenishment, which makes Tat an optimal target for HIV cure and eradication strategies.

These data indicate that therapies targeting extracellular Tat are promising for accelerating proviral DNA decay in cART-treated patients through multiple actions, including (i) blockade of Tat-dependent enhancement of HIV infection in low virus-producing tissue compartments; (ii) the suppression of Tat-induced CD4+ T cell transitioning through a functional cell state primed for latent HIV infection; (iii) inhibition of Tat-mediated cell survival to accelerate the turn-over (replacement) of latently-infected memory CD4+ T cells with uninfected cells; and, iv) relieve of Tat-mediated inhibition of CTL responses. 

Thus, strategies that are aimed at inducing Abs capable of neutralizing the biological activity of extracellular Tat should be pursued for restoring the homeostasis of the immune response as well as to accelerate the rate of CD4+ T cell reservoir decay.

## 5. Anti-Tat Antibodies Protect from Disease Progression

### 5.1. Naturally Occurring Anti-Tat Antibodies

**Figure 5:**
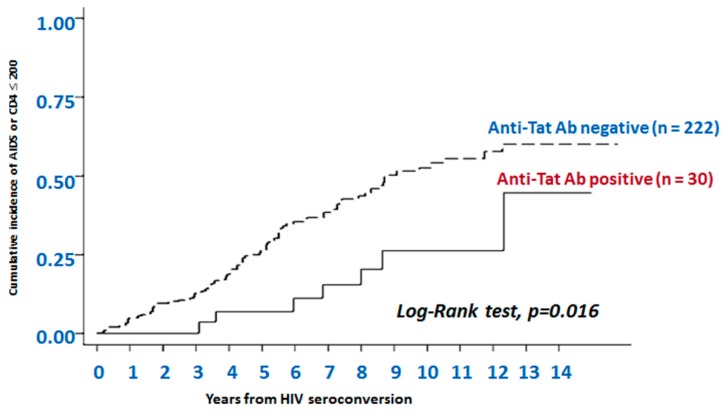
The presence of anti-Tat antibodies is predictive of non-progression to AIDS or AIDS-related events. Data are from 252 HIV+ individuals enrolled in the observational Italian HIV Seroconversion Study conducted from 1985 to 2000. Kaplan-Meier curves show progression to AIDS or to CD4+ T cells count ≤200 cells/μL over 14 years of follow-up in individuals negative or positive for anti-Tat Abs. Anti-Tat Ab positivity: titers ≥100. Subjects were evaluated every three years, stratified by anti-Tat serostatus. The cumulative incidence of AIDS or severe immunodeficiency was calculated for both the anti-Tat Ab positive subjects and the anti-Tat Ab negative subjects; disease progression was significantly slower in the anti-Tat Ab positive subjects than in the anti-Tat Ab negative subjects (*p* = 0.016, log-rank test).

Evidence of the occurrence of anti-Tat Abs in the course of HIV infection was first reported in the late ’80s [110], and it soon became apparent that, unlike Abs against HIV structural proteins, Abs against Tat were only present in a minority of infected individuals [110,111], and that there was an inverse relationship between progression to disease (associated with p24 antigenemia, plasma viral load (VL) and CD4+ T-cell loss) and anti-Tat seropositivity [112,113,114,115]. To gain more insights, a cohort of 252 HIV-1 seroconverters was evaluated retrospectively to assess the relationship between the anti-Tat serostatus and the incidence and risk of progression to disease over an observation time of up to 14 years (median follow-up time: 7.2 years). The risk of progression was found to be lower in anti-Tat Ab-positive subjects as compared to anti-Tat Ab-negative individuals. In particular, no progression was observed in the persistently anti-Tat Ab-positive subjects. However, progression occurred in those who lost anti-Tat Ab-reactivity, and was even faster in the persistently anti-Tat Ab-negative subjects, which suggested a close association between the anti-Tat serostatus and disease progression (Figure 5) [116].

In a subsequent prospective observational study (ISS OBS T-003, ClinicalTrials.gov NCT01029548), 61 asymptomatic individuals naive to cART were followed for up to 42 months (median follow-up time: 24 months) [117]. Of the 20 (32.8%) with anti-Tat Abs, none of the subjects with a persistently high (i.e., above the threshold of positivity for all the three classes of immunoglobulins) anti-Tat Ab response had to initiate cART by the end of the study. In contrast, those that were persistently anti-Tat Ab-negative had to start cART after 17 months, and those with a weak and transient anti-Tat Ab response initiated antiretroviral therapy after 30 months [117], confirming the association found in the retrospective study [116]. Further, individuals seropositive for anti-Tat Abs showed a significant containment of CD4+ T-cell loss and of plasma VL increases, as compared to the anti-Tat Ab-negative subjects, whereas there was no correlation with Abs against Env or Gag [117].

Of importance, as evidenced in another observational study (OBS IFO), the response to cART initiation is also affected by the anti-Tat serostatus. In fact, anti-Tat Ab-positive subjects responded faster (>3 times) and stronger (VL persistently undetectable) to cART initiation than anti-Tat Ab-negative individuals (*p* < 0.0001, Log-Rank test) [118]. Thus, the Ab response to Tat, which infrequently develops in the course of HIV infection, is associated with a slower and milder evolution of the disease and a better responsiveness to antiretroviral therapy, which suggests a protective role of anti-Tat Abs, conceivably due to the neutralization of extracellular Tat. What drives acquisition and loss of anti-Tat Ab response is unknown as yet. It remains that the prevalence of anti-Tat Abs is relatively low (15–33%) in individuals that were infected with HIV-1 clade B, and that those with anti-Tat Abs have a better prognosis (Table 2).

Moreover, a cross-sectional analysis of total and epitope-specific Abs against HIV Tat clade B (Tat B) in samples from Italian, Ugandan, and South African HIV-infected individuals revealed a substantial cross-recognition of Tat B, which was in agreement with the high degree of sequence conservation found in a Tat region critically relevant for its transactivating activity (aa 1–58), as determined by sequencing Tat from viruses circulating in the three countries [119].

### 5.2. Vaccine-Induced Anti-Tat Abs

Based on the previous evidence on the protective role of anti-Tat immunity, Tat was chosen as a vaccine candidate for preclinical and clinical development for the prevention and treatment of HIV-1 infection. The vaccine administered was a subunit vaccine made of recombinant biologically active HIV-1 B Clade (BH10) Tat protein (referred herein as “Tat”) manufactured under GMP for clinical trials. The manufacturing process was specifically developed to prevent oxidation and to keep the protein in its native active form, which is necessary for inducing an effective Ab response against conformational epitopes, which are key for Tat neutralization. In fact, this may account for the lack of protection that was reported by others in nonhuman primate studies, who either deliberately [120,121] or inadvertently [122] employed Tat proteins that were devoid of full biological activity. The Tat vaccine was formulated in isotonic saline buffer with albumin and sucrose as excipients, and stored at −80 °C until administration. Stability studies of GMP considerably indicated a shelf life of more than three years.

#### 5.2.1. Vaccination of Nonhuman Primates with Tat or Tat/Env

Preclinical studies in nonhuman primates were conducted, showing that immunization with the Tat protein or *tat* DNA in cynomologous macaques is safe, elicits a broad and specific immune response, and, most importantly, induced a long-term protection against infection with the pathogenic X4-tropic SHIV89.6P, an SIV that carries the HIV-1 *tat* and *env* genes, which rapidly causes AIDS and death in these monkeys [123,124,125,126,127,128]. In particular, no sign of overt infection (plasma viremia, CD4+ T-cell loss) were detected in nine of the 12 animals that were vaccinated with Tat (protein or DNA), even though a few proviral DNA copies were sporadically found in a few animals. In contrast, all of the controls became overtly infected, with high viral load and steep CD4+ T-cell decline [123,124,125]. Vaccinated and protected monkeys displayed durable anti-Tat memory T cell responses and they did not show signs of systemic infection throughout a 104-week follow-up, even after two boosters with tetanus toxoid, a stimulus that was known to activate CD4+ T cells and to increase virus replication [120]. In contrast, virus persisted and replicated in peripheral blood mononuclear cells and lymph nodes of infected animals, two of which died [127]. Tat-specific Abs, and CD4+ and CD8+ T-cell responses were high and stable only in the animals that controlled the infection, indicating that vaccination with Tat had induced long-term memory Tat-specific immune responses, and had controlled primary infection at its early stages, allowing for long-term containment of virus replication and spread in blood and tissues. When four of the protected monkeys were rechallenged intravenously with a five-fold higher dose (50 monkey infectious dose, MID_50_) of the same SHIV-89.6P, all of them became overtly infected and underwent CD4+ T-cell loss. However, over time, they regained control of infection, as indicated by the statistically significant and long-lasting reduction of viral replication and CD4+ T-cell number restoration in comparison to the control monkeys. This effect was associated with a strong anamnestic response to Tat, while responses to Gag and Env were nearly undetectable [127].

Further, in a subsequent study in which the cynomolgus macaques were primed intramuscularly thrice with the biologically active HIV-1 Tat protein being delivered by anionic microspheres and boosted subcutaneously twice with Tat protein in Alum and then intravenously challenged with SHIV89.6P, control of infection correlated with both Tat-specific T-cell responses and Abs directed against the glutamine-rich (aa 61–75) and the RGD-integrin-binding (aa 71–85) regions of Tat [129].

A protective role for anti-Tat Abs is also suggested by the results of a preclinical study in which cynomolgus macaques were co-immunized with HIV-1 Tat and Env proteins in a complex. The Env was an oligomeric gp140 protein deleted of the V2 region (Env ΔV2) and Chiron provided it (then Novartis, now GlaxoSmithKline, GSK).

**Figure 6:**
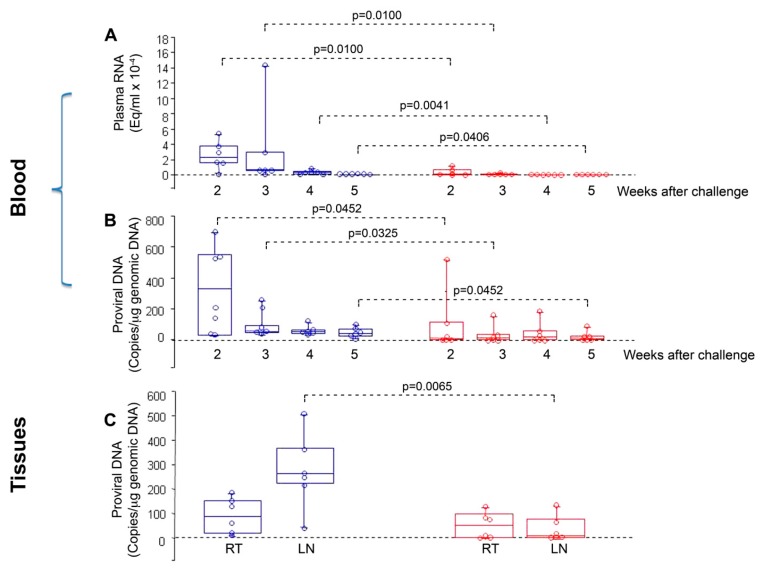
Virological outcome in Tat/Env-vaccinated vs. control monkeys after intrarectal challenge with the SHIVSF162P4cy (70 MID_50_). Box plots of (**A**) viral RNA, (**B**) proviral DNA in blood at 2, 3, 4 and 5 weeks after challenge, respectively; and, (**C**) proviral DNA at week 4 after challenge in rectal tissue (RT) and inguinal lymph nodes (LN). Statistical analysis was performed by the one-sided Wilcoxon rank sum test. Red: monkeys vaccinated with Tat/Env (*n* = 6); blue: control animals (*n* = 6).

In particular, no infection or a statistically significant reduction of viral load and proviral DNA was observed in cynomolgus macaques that were co-immunized with Tat/Env ΔV2 and challenged intrarectally with a high dose (70 MID_50_) of SHIVSF162P4cy [78]. Macaques had been primed twice intranasally with HIV-1 Tat and Env, given together with the LT-K63 mucosal adjuvant, and then boosted subcutaneously with Tat/Env ΔV2 in Alum. No infection or statistically significant lower viral load and proviral DNA were observed in vaccinated monkeys as compared to the controls (Figure 6). Of note, proviral DNA load in the inguinal lymph nodes was significantly lower in vaccinated monkeys as compared to thecontrols, whereas it did not significantly differ in rectal biopsies (Figure 6), which indicated the effective containment of viral infection at the portal of entry, with a block of virus dissemination from the rectum (site of virus inoculum) to lymph nodes following a very high rectal challenge dose [78].

In a similar approach, rhesus macaques were primed mucosally with replicating adenoviral vectors carrying the HIV-1 clade B *tat* and *env* transgenes and boosted systemically with the Tat and Env proteins. Although all macaques became infected upon intravenous homologous challenge with a high dose of SHIV-89.6P, those that were vaccinated with Tat/Env reduced chronic viremia by four logs as compared to the controls (*p* < 0.0001) and did not experience a severe CD4+ T-cell loss. Of note, control of infection correlated with Tat and Env binding Abs [130].

In a further study, the sterilizing immunity or control of infection observed in rhesus macaques immunized with a multi-component vaccine—(multimeric HIV-1 clade C gp160, HIV-1 clade B Tat, and SIV Gag-Pol particles) delivered either systemically or mucosally and challenged orally or intrarectally with SHIV-1157ip, an R5-tropic SHIV encoding a heterologous HIV-C envelope—only correlated with anti-Tat Abs against the N-terminus of Tat [131].

Thus, overall anti-Tat immunity, both humoral and cellular, appears to play a key role in preventing HIV acquisition and disease progression in efficacy monkey models. Further, Tat/Env co-immunization studies also indicate a role of Tat in modulating the immunogenicity of Env towards potentially protective responses.

#### 5.2.2. Phase I Preventive Clinical Trials with Tat or Tat/Env

A randomized, placebo-controlled, double-blinded phase I trial (ISS P-001, ClinicalTrials.gov NCT00529698) was conducted at clinical centers in Italy. Volunteers were healthy, immunologically competent adult volunteers without identifiable risk of HIV-1 infection. The Tat vaccine was safe (primary endpoint) and immunogenic (secondary endpoint) [132,133]. In particular, anti-Tat Abs were induced in all vaccinees (*n* = 14), immunized five times (week 0, 4, 8, 12, 16) either subcutaneously with Alum or intradermally without adjuvant with different doses (7.5, 15, or 30 µg) of the Tat protein. Abs were IgM and IgG, while IgA were detected in 12 of the 14 vaccinees for which immunogenicity could be evaluated. Cellular (lymphoproliferation, YIFN, and IL-4 production) responses to Tat were also induced in the majority of vaccinated volunteers [132]. Of importance, the long-term follow-up (ISS OBS P-001, ClinicalTrials.gov: NCT01024764) showed a persistence of anti-Tat Abs up to five years after the first immunization [132,133].

Based on the promising results from the preclinical study, a phase I preventive trial was also conducted with Tat-Env ΔV2 in Italy (ISS P-002, ClinicalTrials.gov identifier: NCT01441193). This was a multicentric, open label, phase I trial conducted in healthy volunteers, directed to qualify the safety and the immunogenicity of the vaccine based on the association of HIV-1 Tat (7.5 μg) and Env ΔV2 (100 μg) proteins, as compared to vaccination with single proteins. Tat and Env ΔV2 proteins, either in association or as single components, were administered by a prime-boost regimen, consisting of three intradermal priming doses (weeks 0, 4, 8), followed by two intramuscular boosting injections (weeks 24, 36). The Tat/Env ΔV2 vaccination was safe and immunogenic, as indicated by the development of Ab responses to the vaccine antigen(s) in all participants [Ensoli, unpublished data]. Intriguingly, while the highest anti-Tat Ab response was detected, as expected in the volunteers immunized with Tat alone, the highest anti-Env Ab response was detected in those that were immunized with Tat/Env ΔV2, as consistent with the better priming provided by the Tat-mediated entry of Env in DCs [61].

#### 5.2.3. Phase I Therapeutic Clinical Trials with Tat

A therapeutic phase I trial (ISS T-001, ClinicalTrials.gov NCT00505401) was conducted in parallel with the preventive one (ISS P-001), with which it shared the design and the protocol [133,134]. Volunteers were HIV infected asymptomatic individuals, with CD4^+^ T cells/µL ≥400 cells/µL, viral load ≤50.000 copies/mL, and CD4 nadir ≥250, independently from the anti-Tat serostatus at baseline. The study lasted 48 weeks (final analysis) and the volunteers were followed up to five years after immunization. The data indicated the achievement of both the primary (safety) and secondary (immunogenicity) endpoints of the study [133,134]. The Tat vaccine was well tolerated, both locally and systemically, and induced and/or maintained Tat-specific T helper (Th)-1 T-cell responses and Th-2 responses in all subjects with a wide spectrum of functional anti-Tat Abs, rarely seen in HIV-infected subjects. In particular, the administration of biologically active Tat did not increase plasma viremia levels, confirming former data that were obtained in infected macaques showing that bioactive Tat, at the doses used, does not promote viral replication. Conversely, the protein was immunogenic, with anti-Tat Ab titers peaking between week 12 and week 24, to decrease thereafter during the follow-up period. The long-term assessment of the kinetics of anti-Tat Abs (48–144 weeks) revealed that, although decreasing over time, anti-Tat Abs generated by vaccination were still detectable in vaccinees three years after the first immunization. The statistical analysis of the data that were collected up to 48 weeks revealed a significant positive correlation between the levels of circulating CD4^+^ T cells and the anti-Tat IgG or IgA Ab titers in vaccinees, whereas no significant correlation was found for anti-Tat IgM titers. Of note, when the neutralization of Tat-mediated Env entry in DCs by sera from vaccinees or placebo who had remained naive to therapy for the entire follow-up (up to five years) was assessed retrospectively, vaccinees, but not placebo, were found to retain the neutralizing activity, despite that anti-Tat Abs had decreased to barely detectable or undetectable levels. Thus, the neutralization assay appears to be more sensitive than measuring binding Abs by ELISA. Nevertheless, sera from HIV-1 infected individuals negative for anti-Tat Abs by ELISA (placebo) did not neutralize the Tat-mediated Env entry in DCs.

#### 5.2.4. Phase II Therapeutic Clinical Trials with Tat in Italy and South Africa

Following phase I, two phase II studies of cART intensification by the Tat vaccine were conducted, one in Italy and one in South Africa (SA) in patients on successful ART.

The ISS T-002 (Clinicaltrials.gov NCT00751595) was an “exploratory” multicenter, randomized, open label therapeutic trial that was conducted in Italy [92,102]. This trial enrolled 168 HIV-infected (B clade) anti-Tat Ab-negative adults on cART, virologically-suppressed, with CD4+ T-cell counts ≥200 cells/μL. Of those, 155 volunteers were evaluated for immunogenicity, safety, and immunological and virological disease biomarkers after vaccination with 7.5 or 30 µg Tat protein (clade B) without adjuvant, administered intradermally three or five times, one month apart. Both primary (immunogenicity) and secondary (safety) endpoints were met. Of the four vaccine regimens evaluated, the 30 µg given three times was the most effective in inducing anti-Tat Abs Abs [85]. In fact, although anti-Tat Abs were induced in most vaccinees (79%), the highest frequency occurred in the volunteers that were immunized with the 30 μg Tat dose (89%), especially in those receiving three administrations (92%). In contrast, the frequency of Ab responders was 70% in the two arms of the 7.5 μg Tat dose regimen. The Ab response in the 30 μg Tat dose regimen was superior to the 7.5 μg Tat regimen also in terms of strength, as indicated by peak IgG titers, capability of inducing anti-Tat Abs of different isotypes, and durability [92,102].

A nested study was conducted to determine whether the Abs that were induced by the clade B Tat vaccine were capable of recognizing Tat from other clades, a valuable feature when thinking of potential worldwide use. The 38 volunteers that were vaccinated with the 3 × 30 μg Tat dose regimen were chosen because of the optimal anti-Tat Ab response [92]. As for the inclusion criteria, at baseline, they were all negative for anti-Tat B Abs and none of them reacted with any other Tat tested (clade A, C, and D). After Tat vaccination, 33 volunteers (86.8%) mounted an anti-Tat B Ab response. Notably, 24 of these 33 vaccinees (72.7%) developed Abs also recognizing Tat from clade A, C, and D, which represent the vast majority of circulating strains [135]. In particular, four recognized only one additional clade, mostly Tat D (*n* = 3), which is phylogenetically close to clade B, followed by Tat A (*n* = 1). The remaining 20 vaccinees developed anti-Tat Abs crossrecognizing Tat from two (*n* = 11) or three (*n* = 9) non-B clades investigated. As expected, none of the five (13.2%) vaccinees not mounting an Ab response to Tat B cross-recognized Tat from the other clades tested.

Upon vaccination, a durable increase of peripheral blood CD4+ T-lymphocyte counts, CD4:CD8 ratio, B cells (including memory B cells) and NK cell number was observed over three years, as compared to individuals on effective ART alone, who were enrolled in a parallel observational study (ISS OBS T-002) (ClinicalTrials.gov NCT01024556) that served as an external reference group [92,102,136]. The proportion of central memory CD4+ and CD8+ T cells increased, at the expense of the naïve and effector memory counterparts. Functionally, the cellular immune responses against HIV (Env) and common recall antigens [Candida, CEF (CMV, EBV, Flu peptides)] were improved, as compared to the reference group.

Of outmost relevance, Tat immunization induced a reduction of HIV-1 DNA load in blood, especially in volunteers that were receiving Tat 30 μg, given three times. HIV-1 DNA decay was associated with the presence of anti-Tat IgM and IgG and neutralization of Tat-mediated Env entry in DCs, which predicted at 48 weeks the significant HIV-1 DNA reduction starting at year 3. In fact, although the ISS T-002 trial ended at 48 weeks, at study closure follow-up data were available up to 96 weeks for 76 volunteers, and up to 144 weeks for 45 volunteers, which allowed for the detection of the proviral load reduction and prompting us to conduct an observational study (ISS T-002 EF-UP, ClinicalTrials.gov NCT02118168) to extend the monitoring of immunological and virological parameters, including CD4+ T-cell counts and HIV proviral DNA load in vaccinees. The steep reduction of proviral DNA continued over the eight-years follow-up. During this time, anti-Tat Abs persisted in a high proportion of participants, particularly in the Tat 30 µg regimens (49% of the vaccinees) [136]. In fact, as compared to the 7.5 µg regimens, volunteers vaccinated with the 30 µg Tat regimens showed persistent anti-Tat IgG and IgA Abs (log-rank Test *p* = 0.0179 and 0.0128, respectively), and those who developed anti-Tat Abs of all three classes were more likely to maintain the Ab response to Tat (at least one class) [66% vs 23% (two classes) and 27% (one class), Log-Rank Test *p* = 0.0007] [136]. Of note, cellular immune responses to Tat also contributed to the DNA decay, as already observed at three years post-vaccination [102], which is in agreement with epidemiological evidence and data from Tat vaccination studies in non-human primates.

**Figure 7:**
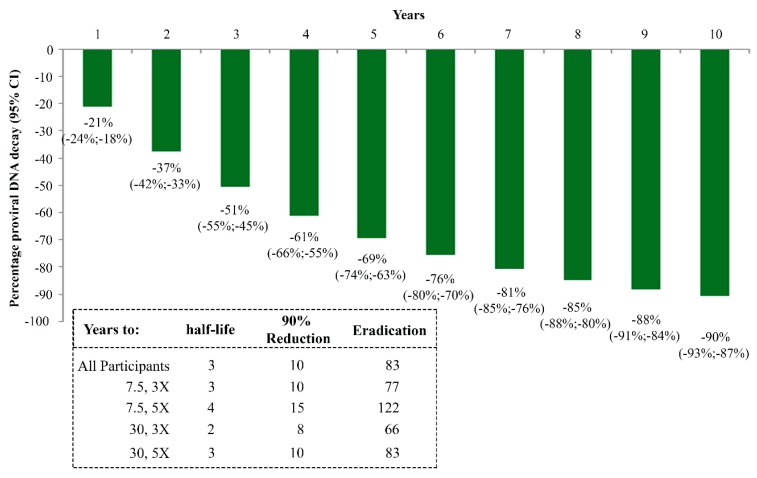
Kinetics parameter estimates of proviral DNA decay in vaccinees stratified by Tat vaccine regimens. Estimates calculated according to Random-effects regression model for decay with first-order kinetics effect, as in Finzi D, et al. Nat Med, 1999 [99]. Estimates of HIV-1 DNA annual decay in all vaccinees expressed as the percentage of HIV-1 DNA decay with 95% confidence interval (upper panel) or years to 50% reduction [half-life (t_1/2_)], to 90% reduction and to eradication for all vaccinees and by Tat vaccine regimens (lower panel) are shown.

Of importance, the proviral DNA reduction that was observed led to an estimated decay over 10 years of 90% (Figure 7), far exceeding the estimates that were calculated in individuals on effective cART only, as reported by others [137,138] (Table 3).

In fact, the estimated half-life of proviral DNA decay diminished to 2–3 years in the Tat immunized individuals, a 4- to 6-fold reduction as compared to subjects on effective cART alone (12 years) (Table 3).

Further, the velocity of decay was fastest (one year) in vaccinees with full virologically suppression (undetectable HIV-1 RNA copies/mL), followed by those (three years) with residual viremia (≥1≤40 RNA copies/mL), while the vaccinees with viremic blips (≥40 RNA copies/mL) experienced the slowest decay (four years) of proviral DNA load in pheripheral blood (Table 3). In individuals on effective cART only, these estimates were similarly affected by the viremic status, but with notable differences. First, the estimated proviral DNA half-lives were much longer than in the vaccinees in all three viremia categories [136] (Table 3). Second, the impact of the viremic status was markedly contained (1–4 years) in Tat vaccinated volunteers, as compared to individuals on effective cART alone (7–22 years), which indicated the important role of anti-Tat immunity in limiting HIV reservoir replenishment. This was the case, even in subjects with viremic blips, in whom a striking reduction of proviral DNA half-life from 22 to four years was estimated when the cART-treated individuals were compared to vaccinees (Table 3).

Based on these results, the 30 µg Tat given intradermally three times four weeks apart was the regimen that was chosen for the confirmatory randomised, double-blind, placebo-controlled, safety, and immunogenicity phase II therapeutic trial (ISS T-003, ClinicalTrials.gov NCT01513135) that was conducted in South Africa (SA) in 200 HIV-infected (clade C) anti-Tat B Ab-negative adults, virologically suppressed, with CD4+ T-cell counts ≥200 cells/μL. Although administered in a population with a different genetic background, infected by different virus subtypes, and treated with different drug regimens, vaccination with clade B Tat was safe and induced durable, high titres anti-Tat Abs of different isotypes [92,102,136,139]. Further, as for the ISS T-002 trial, vaccine induced anti-Tat Abs were capable of cross-clade recognition and neutralization, which correlated with the increase of CD4+ T cells [139], which were a key target for cART intensification. Notably, differently from the Italian trial, 29 of the 100 vaccinees had anti-Tat Abs at baseline directed against Tat C (*n* = 22; 76%), D (*n* = 4; 14%), or A (*n* = 12; 41%) [139]. Tat B vaccination significantly boosted crossclade binding Abs (bAbs) titers in all 29 volunteers. In the remaining 61, vaccination induced both anti-Tat B responses and, to a variable extent, cross-clade bAbs [139]. Importantly, vaccination limited viral load rebound and maintained CD4+ T-cell counts above the baseline levels in non-compliant subjects as compared to placebo, which suggested that Tat vaccine intensification of cART may counterbalance, and hopefully abrogate, the consequences of reduced treatment adherence [139]. So far, no other current treatment has been shown to achieve these effects.

## 6. Conclusions

Overall the data that are presented indicate that Tat is critical in the HIV-1 life cycle, since, when targeted during natural infection, it contains viral replication with no/low progression, while in vaccinated macaques it either prevents overt infection or controls it [123,126,127,128]. This is consistent with the fundamental role that Tat plays in driving effective reverse transcription and transcription from proviral DNA. Although the exact mechanism by which anti-Tat immunity promotes proviral load reduction is unknown, our preclinical and clinical trial results indicate a correlation with the anti-Tat Ab-mediated neutralization of HIV-1 entry in DCs, which suggests the blockade of replenishment of the reservoir as a possible mode of action (Figure 4) [78,102,136]. In fact, the block of Tat-mediated HIV entry into DCs by anti-Tat Ab predicts proviral load decay [102,136]. However, the increasing appreciation of the multifaceted roles that extracellular Tat plays in HIV infection and in disease pathogenesis, reviewed here, suggests that anti-Tat Abs may also promote proviral load reduction by blocking other extracellular Tat activities, especially those that affect the immune system. For example, the reduction of effector memory CD4 and CD8 T lymphocytes (and the concomitant increases of central memory counterparts) that were observed in vaccinees [92] is consistent with the reported effect of extracellular Tat to promote transition of CD4 and CD8 T cells towards an effector phenotype [62,63]. Along the same line, the Tat vaccinees experienced a reduction of inflammatory and immune activation markers that remain elevated, despite suppressive cART [92], which is consistent with the pro-inflammatory and immune activating role of extracellular Tat [52,54,55,56,60,61,62,63,64,69,70,71,72,73,74,76].

In non-human primates, vaccine-induced anti-Tat immunity appears to prevent infection, to confine the virus at the portal of entry or to contain it when systemic spreading occurs [123,126,127,128]. In humans, the epidemiological evidence of protection from disease progression in individuals naïve to therapy [116,117], and the results of proviral DNA decay in subjects on cART developing anti-Tat Abs upon vaccination [102,136] represent the strongest evidence of the key role that Tat also plays in supporting residual disease in virologically suppressed cART-treated individuals. Of note, in all above cases, protection was consistently found to be associated to anti-Tat Abs.

Conversely, the contribution of anti-Tat cellular responses, which are present in most infected individuals, is more difficult to determine, which is mostly due to the inherent difficulties in measuring consistently cellular responses [140,141]. Upon acute infection, CD8+ CTLs are the main contributor to achievement and maintainance of the viral setpoint [142]. Epidemiological data showed that CTLs against Tat are found at high frequency, especially in individuals who spontaneously control the infection (controllers) [143], and the early detection of CTLs against Tat and Rev, but not against Gag, reverse transcriptase, or Nef were associated with delayed progression in individuals naïve to ART [143,144,145]. Upon acute infection, anti-Tat CTLs are readily induced and escaped in macaques [146,147] and in humans, without the apparent loss of Tat activities [148]. Similarly, Tat-based vaccines that aimed at inducing cellular responses alone failed in demonstrating any therapeutic efficacy [149]. This may also explain the failure of a therapeutic vaccine based on a single, presumably linear, universal Tat B-cell epitope [150].

In contrast, Tat is apparently unable to escape Abs, which indicates that structural alterations affecting its conformation or the ability to undergo conformational changes required to interact with host factors also impair its functions. Of note, despite being reported as a very variable protein, Tat is highly conserved in the first 58 aa of exon 1 [119,151], which are critical for the transactivating activity [152], with most of the mutations occurring in the second exon, where the conformational costraints are low and the functions are somewhat “ancillary”, as suggested by its variable length and the existence of functionally competent 1-exon Tat. Moreover, coevolving mutations in functionally distinct domains appear to be compensatory and to maintain Tat functions [153]. This is in substantial agreement with crystallography data, which indicated that, with a few constraints in the first exon, Tat is a poorly structured protein that is capable of standing mutations at several sites without loosing vital functions [154,155], while maintaining the capability of interacting with an extraordinary high number of putative ligands [8,9,10,11,12].

We have not measured the Tat concentrations in our patients’ sera. The main reason is that an ELISA for the detection of Tat in sera or plasma is very cumbersome to set up, due to a very high background signal, and it is not available. However, a few groups reported that serum levels comprised between 0.1 and 40 ng/mL [46,59,156] while using homemade ELISAs, which makes it difficult to compare the results and to draw conclusions. However, the levels of Tat may be much higher in tissues where viral replication occurs (mainly the lymph nodes), than in the serum, as indicated by tissue staining [20]. Further, after release, Tat binds to heparan sulfate proteoglycans [21,35], and most of the Tat that is released by infected cells is trapped and stored in the extracellular matrix (ECM) in a biologically active form, as previously shown [21]. Thus, Tat may be envisaged as a locally acting “cytokine” stored in and released from the ECM as demonstrated for bFGF [20,21], a mechanism that is also hypothesized to occur for chemokines [157].

To date, the most advanced evaluation of the Tat vaccine has occurred in the therapeutic setting due to its several advantages over the preventive setting [158]. In particular, it requires a much smaller sample size, and therefore is much less expensive, while providing a rapid first proof-of-concept of efficacy of a vaccine design as well as biomarkers assessment. It can be conducted in developed countries, where it has a broad application with key potentials in the most affected populations.

In perspective, therapeutic immunization with a Tat vaccine may find applications in different contexts. In the Test & Treat scenario, intensification by the Tat vaccine may accelerate time-to-response to therapy, contain immune system damage and help to reduce reservoir formation and size, which is a prerequisite for achieving a functional cure. In chronically infected individuals on suppressive cART, the Tat vaccine may contribute to further reduce or block residual viremia, thus limiting or solving the low-grade chronic inflammation and immune dysregulation responsible for non-AIDS-related complications. Further, in poor immunological responders, the Tat vaccine can improve CD4+ T-cell recovery. Intensification by the Tat vaccine may also be used to simplify cART and to prevent the risks of suboptimal adherence to therapy, which includes the selection of drug-resistant HIV variants. In this regard, because of the key role of Tat in the virus life cycle, the Tat vaccine may represent an extremely valuable immunotherapy to Figureht multidrug-resistant strains. Eventually, intensification by the Tat vaccine may also lead to a functional cure in chronically infected individuals.

The Tat-Env vaccine appears to be an optimal candidate for preventive vaccination, as indicated by the correlation of the therapeutic Tat vaccine efficacy with neutralization of the Tat-mediated entry of Env into DCs, and the evidence of protection that was observed in preclinical studies. In fact, it is conceivable that Tat-Env co-immunization favors the Tat-mediated entry of Env in DCs, which enhances the priming of anti-Env immune responses.

In addition, as discussed above, most Tat produced is released and it also exerts a plethora of effects on uninfected target cells, as well as on the cells that are not HIV-1 targets, which makes it a major dysregulator of important host systems, such as the immune, vascular, and central nervous system. Thus, the neutralization of extracellular Tat by anti-Tat Abs might also conceivably reduce the residual disease observed in individuals on long-term effective cART, mostly affecting these systems.

## Figures and Tables

**Table 1:** Tat in the HIV life cycle.

	Reference
It is detected in cell-free virion	[18]
It is detected in infected cells prior to virus integration	[19]
It is released extracellularly in the absence of cell death or cell permeability changes	[20,21]
Initiates reverse transcription (RT)	[22]
Increases the rate of transcription	[23]
Promotes transcription elongation	[24]
Contributes in splicing regulation	[25]
Enhances virion infectivity	[26,27]
Amplifies stochastic basal transcriptional fluctuations at the HIV LTR promoter (the Tat circuitry), establishing active or latent infection, or the reactivation of latent HIV	[13,14,15,16,17]

**Table 2:** Clinical benefits associated with the presence of anti-Tat antibodies.

Study Code	Volunteers Number	Status	Results	Potential Clinical Benefit
**ISS OBS** **T-003** [117]	73	naïve to cART	**Stable CD4+ T-cell counts and contained viral load** in anti-Tat Ab positive individuals throughout the study (3 years) **Persistently anti-Tat Ab positive: no progression** or therapy initiation throughout the study (3 years) **Transiently Ab positive**: therapy initiation after **30 months** **Anti-Tat Ab negative**: therapy initiation after **17 months**	Prevention of progression
**OBS-IFO** [118]	29	starting cART	**Faster and persistent virologic response** to cART, in anti-Tat Ab positive as compared to anti-Tat Ab negative individuals	Improved time-to-response to therapy
**ISS OBS** **T-002**	127	on cART	**CD4^+^ T cell increase** upon cART as compared to anti-Tat Ab neg individuals throughout the study (3 years)	Therapy intensification

**Table 3:** Kinetics of proviral DNA decay in Tat vaccinees as compared to similar cohorts of virologically suppressed patients.

Reference Study	[136]	[137]	[138]
HIV reservoir measure	Total HIV DNA	Total HIV DNA	Integrated DNA
cART duration before enrolment	6 years (mean)	≥ 5 years	≥2 years *
Vaccination	Tat vaccine	None	None
cART continuation through the study	Yes	Yes	Yes
**Proviral DNA decay: estimated half-life**			
All patients	2–3 years	12 years	NA
Persistent virological suppression (VL = 0)	1 year	7 years	>7 years
Residual viremia (≥1≤40 RNA copies/mL)	3 years	12 years	NA
Viremic blips (≥40 RNA copies/mL)	4 years	22 years	NA
**Proviral DNA decay: estimated time to eradication**	**Total body reservoir**	**Total body reservoir**	**Total blood reservoir**
Persistent virological suppression (VL = 0)	31 years	NA	>200 years

* With no detectable viremic blips; NA: not available.
